# Preferences of Iranians to select the emergency department physician at the time of service delivery

**DOI:** 10.1186/s12913-021-07183-9

**Published:** 2021-10-25

**Authors:** Dorrin Aghajani Nargesi, Mohammad Hajizadeh, Mohammadhasan Javadi Pakdel, Elham Gheysvandi, Enayatollah Homaie Rad

**Affiliations:** 1 Guilan Road Trauma Research Center, Guilan University of Medical Sciences, Rasht, Iran; 2 Faculty of Health, School of Health Administration, Dalhousie University, Halifax, Canada; 3 Student Research Committee, Guilan Road Trauma Research Center, Guilan University of Medical Sciences, Rasht, Iran; 4 School of Medical Sciences, Khomein Faculty of Medical Sciences, Khomein, Iran; 5 Social Determinants of Health Research Center, Guilan University of Medical Sciences, Rasht, Iran

**Keywords:** Patient preferences, Emergency departments, Willingness to pay, Discrete choice experiment, Iran

## Abstract

**Background:**

Understanding patient preferences in emergency departments (EDs) can provide useful information to enhance patient-centred care and improve patient’s experience in hospitals. This study sought to find evidence about patients’ preference for physicians when receiving services in EDs in Iran.

**Methods:**

In this discrete choice experiment survey, 811 respondents completed the scenarios with 5 attributes, including type of physicians, price of services, time to receive services, physician work experience, and physician responsibility. Analyses were conducted for different social and economic groups as well as for the total population.

**Results:**

This study showed that the willingness to pay (WTP) for being visited by a physician with a high sense of responsibility was 67.104US$. WTP for being visited by an emergency medicine specialist (EMS) was 22.148US$. WTP for receiving ED services 1 min earlier was 0.417US$ and for being visited by 1 year higher experienced physician was 0.866US$. WTP varied across different age groups, sex, health status, education, and income groups.

**Conclusion:**

As the expertise and experience of providers are important factors in selecting physicians in EDs by the patients, providing this information to patients when they want to select their providers can promote patient-centred care. This information can decrease patients’ uncertainty in the selection of their services and improve their experience in hospitals.

## Introduction

As the main entrance of hospitals, emergency departments (EDs) are the first providers of acute care services for the patients [1]. EDs are responsible for providing initial treatment for a wide range of diseases and injuries, and, in many cases, they play a vital role in saving patients’ life. In Iran, these services can be delivered by general physicians (GPs) who are trained to provide emergency medical services or by emergency medicine specialists (EMSs) who are qualified to deliver treatments for many types of emergencies [2].

Changes in disease patterns, increases in hospital costs, technological changes, and increases in patient expectations have brought several new challenges to health systems worldwide. The demand for ED services has been increased dramatically in recent years, and overcrowding of EDs is one of the main challenges of health systems. Patients are now more aware of their rights in terms of how to receive health care services. As a result, they now prefer to choose their health care providers and seek high-quality services [3–5]. Since patients consider services delivered by the specialists to have higher quality than those provided by GPs, access to the EMSs has become one of the expectations of patients admitting to EDs [6, 7].

Although each hospital in Iran has ED, there are not enough EMS graduates in Iran to provide emergency services. Thus, trained GPs provide emergency services in most EDs in Iran [2, 8]. The first cohort of EMSs was graduated in 2005. Although a report has shown that 302 EMSs were available in the country in 2013 [9], there are no accurate statistics about the number of EMSs working in the hospitals. The existing evidence indicates a lack of EMSs in EDs, and GPs are the primary provider of emergency services in Iran. Hospitals in Iran tend to hire GPs to work in ED because the number of trained GPs in Iran is high; as a result, it is less costly to employ and retain GPs in ED in both developed and less developed regions [10, 11].

Several factors affect patient preferences for choosing physicians. Patients prefer to access physicians at a time and place where they require medical emergency services. The distance between the physician’s office and the patient’s residence or work is important in choosing a physician. The cost of medical care is another critical factor influencing the choice of physician. This is especially important when it is costly to have an appointment with the doctor, the patient’s income is low, and the patient does not have insurance coverage [12]. Patients also prefer physicians with more knowledge, skills, and experience.

Health policymakers need to know patients’ preferences regarding the provision of service in EDs in order to provide high-quality services. Identifying the key factors that patients consider in choosing their physicians in ED helps hospital managers invest their resources in areas patients perceive to be important to them. This, in turn, leads to an improvement in the quality of services provided to patients. Using a discrete choice experiment (DCE) – a scientific method for finding the stated preferences of the people – we sought to find evidence about the patients’ preferences in the selection of physicians in ED at the time of receiving services in Iran.

## Methods

### Survey design and data collection

In this mixed-method study, we applied a DCE to find patients’ preferences in ED. We first determined the attributes and their levels from reviewing the literature and interviewing 12 professions (4 EMSs, 5 GPs, and 3 health policymakers) to design the experiment. The participants were interviewed by phone, and they were selected using a snowball sampling method. In this method, each profession nominated the next participant for the interview. In this stage, we identified 29 attributes. The attributes were shared with 17 experts via email for scoring to choose 5 attributes with the highest scores. These experts consisted of 5 GPs, 5 EMSs, 4 health economists, and 3 health policymakers. They were found by searching the Internet and selected based on their resumes. The five attributes with the highest scores were the price of receiving services, physician years of experience, time to receive services, type of physician, and sense of responsibility of the physician. The selected attributes and their definitions and levels are presented in Table 1.

**Table 1:** The attributes and their definition and levels

Attribute	Price (pr)	Experience (exp)	Time (tm)	Physician type (pt)	Responsibility (rsp)
Definition	Price of receiving emergency service by a physician	Physician experience (in years) working in ED	Waiting time to receive ED service	Type of Physician responsible for the treatment of patients	Sense of responsibility and effort of the physician in treating the patient
Levels	Free	2 years	Immediately	General physician	Low
	0.66US$	5 years	15 min	Emergency medicine specialist	Middle
	1.33US$	10 years	30 min		High
	3.33US$	20 years	60 min		
Number of levels	4	4	4	2	3

D-efficiency criteria were used to select the efficient mix of attributes and the choice set for the final experiment. Finally, 12 choice sets were selected with two alternatives. In each choice set, the respondents should select one alternative of GP or EMS for treatment.

The following formula was used to calculate sample size for DCE [13]:

1 $$ n>\frac{500\times c}{t\times a}. $$

Where *n* is the minimum sample size, *c* is the largest number of levels for any of the attributes (4 in the present study), *t* is the number of choice tasks (12 in this study), and *a* is the number of alternatives (2 in the current study). Based on Eq. 1, the minimum sample size needed for the DCE is 83. By including 20 additional samples for every 31 provinces, the minimum sample size was estimated to be 703.

The survey contained questions about respondent’s age, sex, province of residence, living area (urban or rural area), education level, household income. The survey also consisted of a self-perceived visual analogue scale (VAS) question about the household’s financial status, a self-perceived VAS question about the respondent’s health status, and 12 choice sets for preferences of respondents about ED treatment.

An internet-based questionnaire was designed to collect data for the analysis. Internet surveys were found to be an appropriate method for data collection in DCE surveys [12, 14, 15]. After contacting the Iranian Telecommunication Center (ITC) and receiving the permissions for access to the mobile phone database, 2000 phone numbers were selected randomly across the country using a random calculator software available in the ITC database. Of the total 2000 sent text messages, 1610 were received by the users, and the remaining messages were not delivered to the users. Among those who received the text messages, 837 answered the questions completely (response rate = 51.98%). Before starting the survey, the participants were called to provide detailed instructions on participating in the survey. The link to the questions was then sent by a short message panel to participants to complete the survey questionnaire. We included a question in the survey with an obvious correct answer and removed those participants who provided a wrong answer to this question (12 respondents) from the final sample. Additionally, we excluded those participants who answered the questions faster than the regular time of answering (1.5 min) (14 respondents). This yielded a final sample size of 811 respondents, which was more than the estimated minimum sample size for the study.

### Statistical analysis

A random utility approach was used for the calculation of willingness to pay (WTP). Suppose that individual *k* wants to choose between *i* and *j* (GP or EMS) alternatives. The selection is rational and is based on the utility maximization of the individual. Thus, individual *k* will select the alternative *i* over *j* only if:

2 $$ {U}_{ki}\ge {U}_{kj}, $$

where *U* is the expected utility of selection alternative *i* or *j*. The utility value for individual *k* can be specified as:

3 $$ {U}_k={\propto}_1+{\beta}_1{pr}_k+{\beta}_2{\mathit{\exp}}_k+{\beta}_3{tm}_k+{\beta}_4{pt}_k+{\beta}_5{rsp}_k+{\varepsilon}_k. $$

Where *pr*, *exp*, *tm*, *pt*, and *rsp* are attributes discussed in Table 1 and *ε*_*k*_ is the error term of unobserved factors. The levels should be added to the utility model for each attribute. We eliminated adding the levels of each attribute for simplicity. *β* s are the coefficient of the attributes. Since the utility of attributes was not directly observed and only the preferences of each individual were observed (choosing each choice set meant higher utility compared to other choices), the coefficient of each attribute could be estimated by a mixed logistic regression estimator. Price variable was added into the model to calculate WTP for each type of physician (in this formula for GPs). The monetary value of other attributes was calculated using the following formula:

4 $$ WTP\ (pt)=-\frac{\raisebox{1ex}{$\sigma U$}\!\left/ \!\raisebox{-1ex}{$\sigma pt1$}\right.}{\raisebox{1ex}{$\sigma U$}\!\left/ \!\raisebox{-1ex}{$\sigma pr$}\right.}=-\frac{\beta_4}{\beta_1}. $$

Where *pt*1 was the selection of GP, and *β*_4_ was the coefficient of GP attribute and *β*_1_ was the coefficient of *pr*. All financial data were collected in IR.Rials, which converted to US$ with the currency exchange rate of 150,000IR.Rials–1US$. All analyses were conducted using STATA statistical software version 13.1.

## Results

### Descriptive statistics

The average age of participants was 32.21 ± 11.01 years. Of the 811 participants, 518 (63.88%) were females, and 293 (36.12%) were males. 20.73% had high school or less educational attainment, 4.79% had technician degrees, 34.91% had bachelor’s degrees, and the remaining had higher than a bachelor’s degree. The average monthly income was 346.08US$ ± 177.66US$. The average VAS score for self-perceived health status was 8.11 ± 2.09 and for self-perceived financial status was 4.95 ± 2.12.

### Regression results

Table 2 shows the results of mixed logistic regression. The statistically significant coefficient on price variable (*pr* coefficient = − 0.003, *P*-value = 0.034) indicated that losing less money is favourable for the participants. The coefficient on experience (*exp*) was found to be 0.028 and statistically significant (*P*-value< 0.001). This suggested that more experienced physicians are favourable choices among the respondents. The statistically significantly negative coefficient on time to receive services (*tm* coefficient = − 0.013, *P*-value< 0.001) indicated that people like to receive services earlier. Respondents also preferred to receive services from EMS than GPs (coefficient = 0.706, P-value< 0.001). Besides, people preferred to pay more to receive services for physicians with a high (coefficient = 2.138, *P*-value< 0.001) and middle (coefficient = 1.327, *P*-value< 0.001) sense of responsibility.

**Table 2:** Mixed logistic regression results

Choice	Coefficient	Standard Error	***P***-value	95% Confidence Interval
*Mean*				
Price (pr)	−0. 031	0.001	0.034	−0.060 to − 0.0024
Experience (exp)	0.028	0.003	0.000	0.022 to 0.033
Time (tm)	−0.013	0.001	0.000	−0.015 to − 0.012
Physician type (pt)				
General physician (Ref.)				
Emergency medicine specialist	0.706	0.042	0.000	0.623 to 0.788
Responsibility (rsp)				
High	2.138	0.066	0.000	2.008 to 2.268
Middle (Ref.)	1.327	0.045	0.000	1.239 to 1.416
Low				
*Standard Deviation*				
Physician type (pt)				
Emergency medicine specialist	0.829	0.047	0.000	0.738 to 0.921
Responsibility (rsp)				
High	1.102	0.069	0.000	0.966 to 1.237
Middle	0.386	0.084	0.000	0.221 to 0.551
Low (Ref.)				

*Ref*. reference category in the regression

Table 3 shows the WTP of people for service delivery in ED. As shown in the table, people were willing to pay WTP 0.866US$ for a one-year higher physician experience (*P*-value = 0.033). They were also willing to pay 0.417US$ for being visited 1 min earlier (P-value = 0.034). Furthermore, they were willing to pay 22.148US$ for being visited by an EMS instead of a GP. WTP of people for a physician with a high and middle sense of responsibility was 67.10US$ (*P*-value = 0.034) and 41.67 US$ (P-value = 19.770), respectively.

**Table 3:** Willingness to pay (WTP) of people for service delivery in emergency departments

Choice	WTP	Standard Error	***P***-value	95% Confidence Interval
Experience (exp)	0.866	0.407	0.033	0.068 to 1.663
Time (tm)	−0.417	0.197	0.034	−0.804 to − 0.030
Physician type (pt)				
General physician (Ref.)				
Emergency medicine specialist	22.148	10.326	0.032	1.910 to 42.386
Responsibility (rsp)				
High	67.104	31.607	0.034	5.155 to 29.053
Middle	41.667	19.770	0.035	2.919 to 80.416
Low (Ref.)				

*Ref*. reference category in the regression

Table 4 shows WTP for service delivery in the ED among different groups. The mixed logistic regression model has transposed for the ease of comparing WTP of attributes in different groups. As shown in the table, WTP for EMS for respondents with high school or less educational attainment was 6.70US$, while it was 64.27US$ for those having bachelor’s degrees and 26.38US$ for higher educated groups. Nonetheless, only the less-educated WTP was statistically significant. WTP for male EMS was 25.36 US$, whereas it was 19.29US$ for female EMS. WTP for EMS in people with average health status was 8.01 US$. Additionally, WTP for EMS for those aged between 25 and 45 years was found to be 23.63US$. WTP for EMS for the low-income group, self-perceived low financial status, and middle self-perceived financial status was 8.76US$, 9.58US$ and 20.79US$, respectively.

**Table 4:** Willingness to pay (WTP) for service delivery in emergency departments in different groups

Variable	Emergency Medicine Specialist		Time to Receive Services		Physician Experience	
	WTP	Standard Error	WTP	Standard Error	WTP	Standard Error
High school or less education	6.699**	2.960	−0.096**	0.048	0.376**	0.176
Bachelor’s degree	64.272	163.588	−1.710	4.377	2.602	6.626
More than bachelor’s degree	26.377	16.597	−0.373	0.236	0.831	0.519
Males	25.036	16.009	−0.457	0.296	0.999	0.641
Females	19.290	12.754	−0.354	0.238	0.683	0.459
Low health status	5.279	3.402	−0.126	0.082	0.217	0.161
Average health status	8.011**	2.833	−0.179**	0.066	0.306**	0.121
High health status	116.289	323.951	−1.943	5.427	4.415	12.284
Age < 25 years	23.838	24.651	−0.497	0.521	1.174	1.227
Age 25–45 years	23.636*	14.118	−0.409*	0.247	0.871*	0.521
Age > 45 years	15.338	15.955	−0.327	0.346	0.397	0.437
Low income	8.760**	4.039	−0.197**	0.095	0.440**	0.215
High income	40.074	34.734	0.6913	0.603	1.410	1.218
Low financial status	9.585**	4.369	−0.204**	0.096	0.411**	0.196
Middle financial status	20.789*	11.972	−0.358*	0.209	0.799*	0.465
High financial status	−64.888	173.920	1.215	3.239	−2.244	6.039

*Note*: **P-value< 0.05, * *P*-value< 0.10

WTP for receiving services 1 min earlier among the high school or less educational attainment group was 0.096US$. This figure for the average health status group, people aged between 25 and 45 years old, and low-income groups, low and middle self-perceived financial status were − 0.179US$, − 0.409US$, − 0.197US$, − 0.204US$ and − 0.358US$, respectively. In addition, WTP for a physician with 1 year greater experience was 0.376US$ for the high school or less educational attainment group. This figure was 0.306US$, 0.440US$, 0.871US$, 0.440US$, and 0.411US$ for respondents with average health status, aged between 24 and 45 years old, low income and low and middle financial status, respectively.

Table 5 shows the WTP for physicians with a high and middle sense of responsibility in ED in different groups. As shown in the table, WTP for a physician with a high sense of responsibility was 23.403US$ among high school or less educational attainment groups while it was 21.841US$ among those with average self-perceived health status. WTP for physicians with a high sense of responsibility among low income, low financial status, middle financial status was 37.041US$, 33.416US$, and 60.786US$, respectively. Moreover, WTP for physicians with a middle sense of responsibility was 14.023US$ among high school or less educational attainment groups and 14.735US$ among those with average health status groups.

**Table 5:** Willingness to pay (WTP) for physicians with high and medium sense of responsibility in emergency departments in different groups

Variable	High responsibility		Medium responsibility	
	WTP	Standard Error	WTP	Standard Error
High school or less education	23.403**	10.253	14.023**	6.256
Bachelor’s degree	221.245	566.027	137.443	352.324
More than bachelor’s degree	64.338	40.925	39.653	25.527
Males	77.591	50.146	48.446	31.512
Females	51.771	34.560	31.065	20.950
Low health status	17.601	10.784	9.415	5.978
Average health status	21.841**	7.854	14.735**	5.414
High health status	354.081	989.319	213.736	598.014
Age < 25 years	68.680	71.598	48.133	50.450
Age 25–45 years	68.520*	41.423	42.011	25.624
Age > 45 years	59.405	62.338	29.774	31.543
Low income	37.041*	17.262	22.655**	10.652
High income	103.548	90.402	64.059	56.270
Low financial status	33.416**	15.451	18.866**	8.879
Middle financial status	60.786*	35.333	39.900*	23.365
High financial status	− 166.645	444.005	− 100.756	267.622

*Note*: **P-value< 0.05, * *P*-value< 0.10

## Discussion

This study showed that respondents preferred physicians with greater experience and a high sense of responsibility when they use ED services in Iran. They preferred to receive services earlier and from EMSs than GPs. A study in Australia found that people preferred ED clinicians over GPs. They also preferred cheaper services [16]. Preferring less waiting time for ED services was confirmed among seniors [17]. A study conducted by Askari and colleagues in Iran showed that high knowledge, allocating enough time for examination, expressing special attention as the main important factors in selecting physicians. The performance of the specialist physician was found to be the most important reason to select specialists in Tehran, Iran. For emergency care services, people preferred to receive services from a physician instead of a nurse or paramedic in the United Kingdom [18]. This can be because emergency services are not like other routine health services, and people do not have time to seek and find good quality services. As patients do not have enough information about the quality of ED services, their selections are based on their past knowledge. As patients must choose the best providers in a very short period and are aware that EMSs have more ability to provide services, their WTP for services provided by EMSs is higher than the services provided by GPs. Between choosing EMSs (might be with high or low responsibility) and physicians with high responsibilities (GPs or EMs), people select physicians with high responsibilities. This is because patients think that physicians with high responsibilities have more information about their treatments; thus, they face lower uncertainty in receiving their appropriate services [15, 19]. Policymakers must try to decrease this type of uncertainty and provide necessary information on the performance indicators of physicians to patients to help them to make an informed selection of a physician. These indicators should include the structural, process, and outcome indicators of medical service providers. This information system allows patients to seek out physicians and make informed choices by comparing physician’s performance.

A study in northern England found that people prefer less waiting time and less traveling time for emergency services [20]. A study in the United States suggested that people choose insurance providers that cover the services provided by their physicians. In addition, they are willing to pay 45US$ more to choose those providers that allow visiting the physician 3 days sooner; however, these findings may not be generalizable to ED services [21]. Another study investigated public care services preferences and showed that people preferred their own physicians as they know them and trust the services delivered by them [22].

This study had some limitations. First, although it has been shown that internet-based surveys are appropriate in DCE studies, using internet-based platforms might limit the accessibility of the survey to low-income and less-educated groups in the population. Thus, future face-to-face surveys can provide further understanding about patient preferences in EDs. Second, although we tried to have a detailed discussion with participants on how to answer the questions, the scenarios included in this study might be hard to complete for some participants. To address this limitation, we included a question with an obvious correct answer to identify and exclude participants who did not understand the survey questions. Third, since there are differences in payments for ED services in different countries, the results of this study might not be generalizable to other countries. As different types of health insurances and complementary health insurances are the intermediaries between the patients and the health providers, future DCE studies investigating patients’ selections under the presence of health insurances can provide further insight into patient’s preferences.

## Conclusion

Our study suggested that patients’ WTP for physicians in EDs was inversely related to the cost of services (especially among the poor financial status people) and time to receive services, but also directly related to physicians’ experience, expertise, and sense of responsibility. As the expertise and experience of providers are important factors in selecting physicians in ED by the patients, providing this information to patients can help patients to make an informed selection when selecting their physicians. This, in turn, can improve patient-centerd care in EDs. Availability of high experience physician and health insurance coverage to reduce the cost of services can also improve patients’ experience using health care services in EDs. In addition, as participants are WTP more to receive services from EMS rather than GPs, training and retaining physicians with expertise in emergency medicine is highly recommended.

## Data Availability

The datasets used and analyzed in the study are available from the corresponding author on reasonable request.

## Abbreviations

DCE: Discrete choice experiment
ED: Emergency department
EMS: Emergency medicine specialists
GP: General physician
ITC: Iranian Telecommunication Center
VAS: Visual analogue scale
WTP: Willingness to pay

## References

[1] He J, Hou X-Y, Toloo S, Patrick JR, Gerald GF (2011). Demand for hospital emergency departments: a conceptual understanding. World J Emerg Med.

[2] Ravaghi H, Nasiri A, Takbiri A, Heidari S (2020). Status, role, and performance of emergency medicine specialists in Iran: A qualitative study. J Educ Health Promot.

[3] Pines JM, Mullins PM, Cooper JK, Feng LB, Roth KE (2013). National trends in emergency department use, care patterns, and quality of care of older adults in the United States. J Am Geriatr Soc.

[4] Kellermann AL (2006). Crisis in the emergency department. N Engl J Med.

[5] Comelli I, Scioscioli F, Cervellin G (2020). Impact of the COVID-19 epidemic on census, organization and activity of a large urban emergency department. Acta Bio Medica: Atenei Parmensis.

[6] Abbasi AA, Khan S, Ameh V, Muhammad I (2021). The impact of GP referrals on overcrowding in emergency departments and acute medicine. Br J Healthc Manag.

[7] Keikavoosi Arani L, Ramezani M, AbedinSalimAbadi P (2014). Codification of national accreditation standards for management and leadership in hospitals of Iran. J Mazandaran Univ Med Sci.

[8] Farahmand S, Karimialavijeh E, Vahedi HSM, Jahanshir A (2016). Emergency medicine as a growing career in Iran: an internet-based survey. World J Emerg Med.

[9] Iranian House of Emergency Physician. Emergency physician specialists of Iran. Tehran: 1, 2018. Available from: https://www.ihem.ir.

[10] Chimeh EE, Behbahani AA. Factors affecting the service delivery locations of newly graduated Iranian general practitioners. Iran Red Crescent Med J. 2017;19(2):e40032.

[11] Amiresmaili M, Khosravi S, Feyzabadi VY (2014). Factors affecting leave out of general practitioners from rural family physician program: a case of Kerman, Iran. Int J Prev Med.

[12] Determann D, Lambooij MS, Steyerberg EW, de Bekker-Grob EW, de Wit GA (2017). Impact of survey administration mode on the results of a health-related discrete choice experiment: online and paper comparison. Value Health.

[13] de Bekker-Grob EW, Donkers B, Jonker MF, Stolk EA (2015). Sample size requirements for discrete-choice experiments in healthcare: a practical guide. Patient.

[14] Homaie Rad E, Hajizadeh M, Yazdi-Feyzabadi V, Delavari S, Mohtasham-Amiri Z (2021). How much money should be paid for a patient to isolate during the COVID-19 outbreak? A Discrete Choice Experiment in Iran. Appl Health Econ Health Policy.

[15] Ryan M, McIntosh E, Dean T, Old P (2000). Trade-offs between location and waiting times in the provision of health care: the case of elective surgery on the Isle of Wight. J Public Health Med.

[16] Harris P, Whitty JA, Kendall E, Ratcliffe J, Wilson A, Littlejohns P, Scuffham PA (2015). The Australian public's preferences for emergency care alternatives and the influence of the presenting context: a discrete choice experiment. BMJ Open.

[17] Arendts G, Howard K, Rose JM (2011). Allocation decisions and patient preferences in emergency medicine. Emerg Med J.

[18] Gerard K, Lattimer V, Turnbull J, Smith H, George S, Brailsford S, Maslin-Prothero S (2004). Reviewing emergency care systems 2: measuring patient preferences using a discrete choice experiment. Emerg Med J.

[19] Narayanan S, Chintagunta PK, Miravete EJ (2007). The role of self selection, usage uncertainty and learning in the demand for local telephone service. Quant Mark Econ.

[20] Bhattarai N, Mcmeekin P, Price CI, Vale L (2019). Preferences for centralised emergency medical services: discrete choice experiment. BMJ Open.

[21] van den Broek-Altenburg EM, Atherly AJ (2020). Patient preferences for provider choice: a discrete choice experiment. Am J Manag Care.

[22] Seghieri C, Mengoni A, Nuti S (2014). Applying discrete choice modelling in a priority setting: an investigation of public preferences for primary care models. Eur J Health Econ.

